# Retraction: The Collective Effect of MIP-3α and FL Promotes Dendritic Cell Function Within the Immune Microenvironment of Murine Liver Cancer

**DOI:** 10.3389/fonc.2021.744389

**Published:** 2021-08-23

**Authors:** Frontiers Editorial Office

**Affiliations:** Frontiers Media SA, Lausanne, Switzerland

The Journal retracts the 26 March 2021 article cited above for the following reasons provided by the Authors:

Following publication, concerns were raised regarding the integrity of the images in the published figures. The authors failed to provide a satisfactory explanation during the investigation, which was conducted in accordance with Frontiers’ policies.

This retraction was approved by the Chief Editors of Frontiers in Oncology and the Chief Executive Editor of Frontiers. The authors agreed to this retraction.

